# Endodontic Treatment of Apical Periodontitis Mitigates Systemic Inflammation and Restores the Blood–Brain Barrier Integrity in an In Vitro Model

**DOI:** 10.1111/iej.70216

**Published:** 2026-07-02

**Authors:** Loredana Bergandi, Mario Alovisi, Elena Tachis, Nicola Scotti, Andrea Baldi, Iris Chiara Salaroglio, Sabrina Digiovanni, Lorena Charrier, Francesca Silvagno, Damiano Pasqualini

**Affiliations:** ^1^ Department of Oncology University of Turin Turin Piedmont Italy; ^2^ Department of Surgical Sciences, Dental School, Endodontics Unit University of Turin Turin Piedmont Italy; ^3^ Molecular Biotechnology Centre Guido Tarone University of Turin Turin Piedmont Italy; ^4^ Department of Public Health and Pediatrics University of Turin Turin Piedmont Italy

**Keywords:** apical periodontitis, barrier integrity, cellular permeability, inflammation

## Abstract

**Aim:**

A growing body of research supports an association between periapical inflammation and an increased risk of various systemic effects. However, there is currently no scientific evidence demonstrating a causal relationship between this inflammation and changes in epithelial or endothelial permeability in patients with apical periodontitis (AP). This study aimed to evaluate the potential association between AP, circulating inflammatory cytokines known to affect cellular permeability, and serum proteins associated with an impaired cellular barrier permeability. Additionally, we investigated the effect of endodontic treatment on in vitro endothelial barrier integrity.

**Methodology:**

A total of 27 patients with AP and 27 healthy control subjects were enrolled. AP patients underwent root canal treatment and were followed up at 6 and 12 months post‐treatment. Serum levels of human ZO‐1, Claudin‐5, and VE‐cadherin were measured using enzyme‐linked immunosorbent assays (ELISA). An in vitro model of the microvascular endothelial blood–brain barrier (hCMEC/D3 cells) was used to assess the biological activity of patient sera on barrier function and cellular permeability.

**Results:**

At baseline, patients with AP showed significantly higher serum levels of ZO‐1 and Claudin‐5 compared with controls (*p* < 0.001), whereas VE‐cadherin levels did not differ significantly between groups. Serum ZO‐1 and Claudin‐5 levels progressively decreased at 6 and 12 months following root canal treatment. In adjusted analyses, the baseline between‐group differences for ZO‐1 and Claudin‐5 remained significant after adjustment for BMI and smoking status. ZO‐1 and Claudin‐5 levels showed moderate correlations with IL‐1β and IL‐6 in the AP group at baseline. In vitro, exposure to baseline AP sera increased endothelial permeability, indicating barrier disruption, while sera collected after treatment did not impair barrier integrity. Moreover, cellular ZO‐1 expression remained stable in cells treated with control sera but was markedly reduced after exposure to sera from untreated AP patients (t0), whereas post‐treatment sera (t6 and t12) restored ZO‐1 expression to control levels.

**Conclusions:**

Systemic inflammation in patients with apical periodontitis to deranged endothelial barrier function. Successful endodontic treatment was associated with reduced levels of inflammatory cytokines and the restoration of serum and cellular proteins essential for barrier integrity.

## 1 Introduction

Apical periodontitis (AP) is a common oral disease characterized by periodontal inflammation resulting from an odontogenic infection, ultimately leading to bone loss (Luo et al. 2022). AP, as an immune response to endodontic and periradicular infection, presents significant similarities with chronic periodontitis. Indeed, in both diseases, the persistence of the infective etiological factor determines a shift from a protective role of the immune system to harmful effects on the alveolar bone (Peddis et al. 2019).

AP is no longer considered only a tissue‐limited pathology. Indeed, lipopolysaccharide (LPS), a component of the outer membrane of Gram‐negative bacteria that cause AP, interacts with Toll‐like receptor 4 (TLR4) and co‐receptor CD14 through the bacterial lipopolysaccharide‐binding protein (LBP) on macrophages, neutrophils (Page et al. 2022) and platelets (Brousseau‐Nault et al. 2017) surface. Stimulation of TLRs leads to the activation of signalling pathways that modulate inflammatory gene expression, which is crucial for the innate immune response to inflammation (Kim et al. 2016).

The increased synthesis of pro‐inflammatory cytokines entering the systemic circulation, induce or perpetuate a chronic systemic inflammatory state (Milojevic et al. 2024), leading to systemic immune dysregulation (Ye et al. 2023). Systemic inflammation disrupts epithelial and endothelial barriers through different mechanisms, including cytokine‐mediated junctional breakdown, oxidative stress, cytoskeletal contraction, protease activity, and leukocyte transmigration resulting in increased cellular permeability (Mehta and Malik 2006). This contributes to the development and progression of multiple inflammatory disorders (Bains and Bains 2018); (Caplan et al. 2006); (Joshipura et al. 2006); (Bordagaray et al. 2024; Jakovljevic et al. 2023; Katz and Rotstein 2021; Lamba et al. 2023). It is currently unexplored whether untreated apical periodontitis, leading to inflammatory and pathological systemic effects, may also compromise the epithelial and endothelial barrier integrity, particularly the blood–brain barrier. These barriers depend on tight junctions (TJs) and adherens junctions (AJs) to regulate paracellular permeability and maintain tissue homeostasis (Richards et al. 2022). Pro‐inflammatory cytokines can disrupt these junctions, increasing barrier permeability and enhancing exposure to luminal antigens, thereby amplifying the inflammatory response. Indeed, pro‐inflammatory cytokines, including IL‐1β and TNF‐α, are known to disrupt intestinal barrier integrity by disassembling the TJ (Wells et al. 2017) and also to play a role in cerebral barrier dysfunction (Capaldo and Nusrat 2009).

Notably, serum levels of zonula occludens‐1 (ZO‐1) have been reported in several gastrointestinal and non‐gastrointestinal disorders associated with increased intestinal permeability due to tight junction dysfunction (Fan et al. 2023). Claudin‐5, another critical component of TJ, is also associated with altered cerebral endothelial permeability in different inflammatory bowel and neurological disorders, including depression, insomnia, and some neurodegenerative diseases (Boncuk Ulaş et al. 2023); (Fan et al. 2023). Moreover, increased circulatory levels of the vascular endothelial cadherin (VE‐cadherin), an AJ protein expressed in endothelial cells, have been associated with the inflammation‐based pathogenesis of vasculitis, coronary atherosclerosis, and diabetic retinopathy (Chen et al. 2014).

These findings further support the hypothesis that chronic local inflammation, as observed in AP, can have far‐reaching systemic effects. Previous studies have demonstrated that AP may promote inflammation‐driven insulin resistance in an in vitro model, where root canal treatment may ameliorate inflammatory mediators in vivo and the cellular response to insulin in vitro (Multari et al. 2024). Notably, a prospective cohort study indicates that type 2 diabetes mellitus, particularly when poorly controlled, is associated with delayed periapical healing and less favourable outcomes after root canal treatment, while endodontic therapy, in turn, contributes to a reduction in systemic inflammatory burden and a modest improvement in glycemic control (Davidović et al. 2024). Moreover, AP has been associated with early endothelial dysfunction, already known to be related to both systemic inflammatory diseases and coronary risk factors (Bergandi et al. 2019). Data suggest that the treatment of apical lesions improves endothelial dysfunction since 2–12 months after treatment, and that increased serum levels of endothelin‐1 (a potent endogenous vasoconstrictor), intercellular adhesion molecules (ICAM‐1/CD54) and leukocyte (E‐selectin) of patients with AP return to be completely comparable to those of subjects without apical periodontal disease, as well as the levels of inflammatory markers including IL‐1 and the soluble form of CD14 (sCD14) (Bergandi et al. 2019).

In this scenario, the inflammation triggered by AP could also contribute to modifying epithelial and endothelial permeability, potentially evoking different systemic effects. The activation of endothelial cells, together with the loss of endothelial barrier function, is a hallmark of overshooting immune responses during systemic inflammation (Hellenthal et al. 2022).

Based on our previous data (Multari et al. 2024), demonstrating that baseline serum levels of the pro‐inflammatory cytokines IL‐1β, IL‐6, and IL‐8 were significantly higher in the AP group than in controls after adjustment for smoking and that cytokine levels decreased significantly at 6 and 12 months after treatment compared with baseline, this study aimed, firstly, to evaluate any association between AP circulating inflammatory cytokines and proteins associated with endothelial barrier permeability, as well as to examine the impact of endodontic treatment on these proteins. Additionally, we sought to assess the effect of endodontic treatment on an in vitro cellular model of blood–brain barrier permeability to provide further evidence of restored barrier function.

## 2 Methods

### 2.1 Study Population

This observational study has been written according to the Preferred Reporting items for Observational studies in Endodontics (PROBE) 2023 guidelines. The observational study was designed and carried out with the approval of the *Città della Salute e della Scienza Ethical Committee* (prot. nr. 0094485; CS2/510 and Clinical Trial Registration no. NCT07292363) and conducted in accordance with the latest update of the Helsinki Declaration (World Medical Association 2013). Each enrolled subject provided a signed, informed written consent to participate in the study (informed consent was obtained from the parents of patients under 18 years of age).

For the current research, the authors chose to use the same cohort that they had used for their previous study (Multari et al. 2024), maintaining the same inclusion and exclusion criteria and the relative data analysis of serum inflammatory cytokines. The current analyses were exploratory within a new research project and were not pre‐specified in the original study design, aiming to further investigate the pathological consequences of systemic inflammation driven by AP and to unveil the molecular mechanisms involved in the outcome of endodontic therapy. The study sample was derived from the same prospectively followed cohort used in our previous investigation on systemic inflammatory cytokines in AP. In that parent study, a sample size of 27 subjects per group was estimated to provide 80% power to detect significant differences in IL‐1 levels between AP patients and controls at baseline and between baseline and 1‐year post‐treatment among AP patients, assuming a two‐sided alpha error of 5% and a 20% loss to follow‐up. The present work should therefore be considered a secondary exploratory analysis of that previously established cohort, and no dedicated a priori sample size calculation was performed for the permeability‐related proteins assessed here.

Twenty‐seven consecutive Caucasian, dentate subjects between 14 and 55 years old, with no medical history of diabetes, systemic, oncologic or immune system diseases who were referred to the Endodontic Department of a dental hospital in Northen Italy with a diagnosis of pulp necrosis and AP were enrolled in the study (AP group) and scheduled for primary root canal treatment (RCT) at the time of admission (baseline, t0). All patients receiving medication or with signs or symptoms of generalised periodontitis or other oral pathologies, and those who had received other dental treatments in the 6 months before enrolment, were excluded due to the role played by periodontitis in modulating systemic inflammation (Hegde and Awan 2019).

Healthy, age‐matched control subjects (control group) were randomly selected from a general medical database from the same district. This was designed to ensure matching between cases and controls based on the matching variable (under and over 36 years old). The same inclusion and exclusion criteria adopted for the AP group were applied to control participants, including absence of systemic or inflammatory disease, medication use, generalised periodontitis or other oral pathologies, and no dental treatment within the previous 6 months. Absence of apical periodontitis in control subjects was confirmed at enrolment through both clinical and radiographic examination. Clinical assessment included percussion, thermal and electric pulp testing to verify pulpal vitality, while standardised periapical radiographs confirmed the absence of periapical lesions. These assessments were also performed in cases of suspect AP, such as history of trauma, tooth discoloration, negative response to pulp sensitivity testing, and positive response to percussion test.

Regarding the patients’ health status during the follow‐up period, no major intercurrent illnesses or acute inflammatory events were recorded that could have significantly influenced the investigated proteins.

The diagnosis of AP was clinically assessed for each tooth using percussion, thermal, and electric pulp tests (Diagnostic Unit, Sybron, Orange CA, USA). Radiographic evaluation was performed with digital periapical X‐rays using PSP imaging plates (Dürr, Baden‐Württemberg, Germany) and Rinn XCP positioning devices (Rinn Corp, Elgin, IL, USA). Radiographic assessment was performed using standardised digital periapical radiographs acquired with the paralleling technique and Rinn XCP positioning devices to ensure reproducibility. All radiographic examinations were evaluated by a single experienced endodontist. Diagnosis was established through combined clinical and radiographic criteria routinely adopted for apical periodontitis diagnosis, and teeth which showed radiographic loss of lamina dura and periodontal ligament enlargement > 2 mm at the largest diameters were included in the AP group.

All root canal treatments (RCTs) were carried out by a single experienced operator, following the highest quality standards as recommended by the European Society of Endodontology (European Society of Endodontology 2006). Following local anaesthesia and rubber dam isolation, access cavity preparation was performed. Canal scouting was initiated manually using a #10K‐file. Mechanical glide path and canal shaping were completed to working length using ProGlider and ProTaper Next rotary systems (Dentsply Sirona) with an endodontic motor set to the manufacturer's recommended parameters. Working length was determined electronically (Root ZX, J. Morita Corp., Kyoto, Japan) and confirmed radiographically. Irrigation was performed by alternating 5% sodium hypochlorite (Niclor 5, OGNA, Muggiò, Italy) and 10% EDTA (Tubuliclean, OGNA, Muggiò, Italy) for a minimum of 30 min (Haapasalo et al. 2014). Upon completion of canal shaping, each canal was dried using sterile paper points. Root canal obturation was performed with gutta‐percha points and either a zinc oxide‐eugenol (ZOE) sealer (Pulp Canal Sealer EWT, Kerr Endodontics, Orange, CA, USA) or a bioceramic sealer (Total Fill, FKG, La Chaux‐de‐Fonds, Switzerland; AH Plus, Dentsply Sirona, Italy). A temporary restoration was placed following obturation. Patients were discharged with a prescription for analgesics, if needed and scheduled for placement of a permanent restoration within 2 weeks.

All the enrolled subjects were recalled for 6‐month (t6) and 12‐month (t12) post‐treatment follow‐up visits, and for an additional blood sample collection. Clinical and radiological examinations were also performed at each follow‐up visit. The healing patterns of the LEOs were assessed according to the criteria proposed by Friedman (Friedman and Mor 2004), and each clinical case was classified as healed, healing, or not healed. The control group was also followed up at 6 and 12 months. As there were no clinical or radiographic signs of periapical lesion development and no changes in lifestyle and medical history of these patients, this group was considered unchanged during the study and representative of a healthy population, except for one subject who was excluded as he developed AP.

This study was conducted exclusively using fully anonymized data, and no identifiable patient information was accessed. Investigators, medical and laboratory personnel, data analysts, and outcome assessors were all blinded to the origin of the samples throughout every stage of the study.

### 2.2 Blood Sample Collection

At the time of enrollment (0 months), at 6 and 12 months after non‐surgical RCT for the AP patients (AP group) and also for all subjects (control group), blood samples were drawn into serum separator tubes (BD Vacutainer; Bectin Dickinson Biosciences, Franklin Lakes, NJ, USA). After clot formation, sera were isolated by centrifugation for 10 min at 1000× *g* and immediately frozen at −80°C before being analysed. Hemolyzed samples were rejected, and a further withdrawal was made.

### 2.3 Enzyme‐Linked Immunosorbent Assay

The levels of human ZO‐1, Claudin‐5 (Bio‐Techne, Minneapolis, MN, USA), and VE‐cadherin (R&D Systems, Minneapolis, MN, USA) were measured by enzyme‐linked immunosorbent assays (ELISA, R&D Systems, Minneapolis, MN, USA), following the manufacturer's instructions. Intra‐assay and inter‐assay coefficients of variation were all < 6%. All samples were measured in duplicate. The amounts of the analytes were expressed as pg/mL (ZO‐1 and Claudin‐5) or ng/mL (VE‐cadherin) of serum.

### 2.4 Cell Cultures and Treatments

The immortalized hCMEC/D3 cells, a human brain microvascular endothelial stabilized cell line that retains blood–brain barrier (BBB) properties in vitro, were a kind gift from Prof. Pierre‐Olivier Couraud (Institut Cochin, Centre National de la Recherche Scientifique UMR 8104, INSERM U567, Paris, France) (Weksler et al. 2005).

Cells were seeded at 50000/cm^2^ density and grown for 7 days up to confluence in a 6‐well plate for immunofluorescence and in a Transwell insert surface 0.33 cm^2^ (Corning Life Sciences, NC, USA) for TEER measurements and permeability assay to allow the formation of a competent BBB. Cells were cultured in EndoGRO Basal Medium (Lonza, Basel Switzerland), 0.2% EndoGRO‐LS Supplement, 5 ng/mL recombinant human (rh)EGF, 50 μg/mL ascorbic acid, 10 mM L‐glutamine, 0.5 μM hydrocortisone hemisuccinate, 0.75 U/mL heparin sulphate, 2% fetal bovine serum (FBS; all from Lonza), as previously described (Salaroglio et al. 2018). Cells were treated for 24 h with sera diluted 1:3 in growth medium for the following specific assays.

### 2.5 Permeability Across the BBB Monolayer Assay and Transendothelial Electrical Resistance (TEER)

The permeability to dextran 70‐fluorescein isothiocyanate (DEXT) across the BBB monolayer was used to measure TJ integrity, as previously described (Salaroglio et al. 2018). DEXT normally does not cross the BBB. When the hCMEC/D3 cells reached confluence, they were incubated for 48 h with each serum from the AP or control group at 0 months, and at 6 and 12 months after endodontic treatment. As a negative control, hCMEC/D3 cells were grown for 48 h in DMEM medium, and as a positive control of inflammation cells were treated with rhIL‐6 (#ab119444; Abcam, Cambridge, UK; 200 pg/mL). At the end of the incubations, the culture medium was replaced in both chambers. 2 μM 70 kDa dextran‐FITC was added to the upper chamber of the Transwell. After 3 h the medium in the lower chamber was collected. Cells were washed twice with PBS, sonicated and resuspended in 300 μL PBS. A 50 μL aliquot was used to measure the protein contents (BCA kit, Merck, Milan, Italy). The amount of 70 kDa dextran‐FITC was measured fluorimetrically, using a Synergy HT microplate spectrofluorimeter (Bio‐Tek Instruments, Winooski, VT, USA). Excitation and emission wavelengths were: 494 and 518 nm. Fluorescence was converted in nmol/cm^2^, using a calibration curve previously set. The autofluorescence of the medium, considered as a blank, was subtracted from each measure. The permeability coefficients were calculated according to (Siflinger‐Birnboim et al. 1987). The DEXT amount was expressed as nanomoles/mg cell proteins, according to the titration curves prepared in each experiment.

TEER was measured using a Voltohmetro Millicell‐ERS (Millipore, Bedford, MA, USA), according to the manufacturer's instructions, after 24 h of incubation with each serum from the AP or control group at 0 months, and at 6 and 12 months after endodontic treatment. The mean TEER value of the plastic insert in the absence of cells was 26.73 ± 0.45 Ω cm^2^ (*n* = 8). This value was subtracted from each value obtained in the presence of the cells.

### 2.6 Fluorescence Microscopy

For the confocal microscopy study, cells were seeded at 4 × 10^4^ cells/chamber well and were incubated at 37°C in a humidified 5% CO_2_ atmosphere. After 24 h, the medium was removed, and the cells were washed three times with PBS. Finally, cells were fixed with 4% paraformaldehyde in PBS for 15 min at room temperature and permeabilized with 0.1% Triton X‐100 in PBS for 5 min at room temperature. Cells were incubated o.n. with anti‐Claudin‐5 mAb (clone 4C3C2, #35‐250, Invitrogen Life Technologies, Carlsbad, California, USA, 1:50) and ZO‐1 mAb (clone 1A12, #33‐9100, Invitrogen Life Technologies, 1:70) followed by an Alexa Fluor 488‐conjugated goat anti‐mouse IgG (H + L) (**#**A28175, Life Technologies, 1:200) and Alexa Fluor 633‐conjugated goat anti‐Mouse IgG (H + L) (**#**A‐21052, Life Technologies, 1:200), respectively, for 1 h at room temperature. DAPI was used for nucleus staining. Samples were kept at 4°C in the dark until microscopic examination. Confocal acquisitions were performed by using a 20× objective (bar scale: 100 μM). For each experimental point, a minimum of five microscopic fields were examined.

### 2.7 Statistical Analysis

All continuous data are reported as mean ± standard deviation (SD) or as median with interquartile range (IQR), as appropriate. The Shapiro–Wilk test was used to assess normality. Categorical variables are presented as absolute frequencies and percentages.

Comparisons of continuous variables between the AP group and the control group were performed using either an unpaired Student's *t*‐test or a Wilcoxon rank‐sum test, depending on data distribution. Intra‐group differences between baseline and follow‐up were assessed with a paired Student's *t*‐test or a Wilcoxon signed‐rank test, as appropriate. For categorical variables, comparisons between groups were carried out using the chi‐square test.

For the main investigated proteins with non‐normal distributions, additional between‐group analyses at baseline and for 12‐month change scores were performed using bootstrap quantile regression models to estimate median differences, with group as the main predictor and BMI and smoking status as covariates. Change scores were calculated as the difference between the 12‐month and baseline values. For within‐group longitudinal comparisons of the serum protein levels, prespecified pairwise comparisons were performed between baseline and 6 months and between baseline and 12 months; Bonferroni correction was applied for these two comparisons, with statistical significance set at *p* < 0.025. Effect sizes for non‐parametric comparisons were calculated as *r* = |*z*|/√*N*, where *z* is the standardised test statistic, and *N* is the number of observations included in the analysis.

Correlations between continuous variables were evaluated using Pearson's or Spearman's correlation coefficients, as appropriate. Because 12 correlation analyses were performed, Bonferroni correction was applied, yielding a significance threshold of *p* < 0.004.

All statistical analyses were performed using a two‐sided significance level of 5% and Stata version 18 (StataCorp LLC, College Station, TX, USA).

## 3 Results

Based on the published results (Multari et al. 2024), no significant differences were observed in demographic variables between the AP and control groups (age, *p* = 0.63; gender, *p* = 0.402). Moreover, patients with AP showed higher baseline serum levels of IL‐1, IL‐6, IL‐8, and TNF‐α compared with control subjects, with cytokine levels decreasing by RCT. In this context, the present study analysed the modulation of vascular permeability and its possible correlation with cytokine levels.

Firstly, we investigated the possible inflammation‐associated modulation of serum levels of TJ proteins like ZO‐1 and Claudin‐5, as well as VE‐cadherin, a protein contained in the AJ, in AP patients before and after 6 and 12 months of treatment, and in the control group. At baseline, serum ZO‐1 and Claudin‐5 levels were significantly higher in the AP group than in the control group, whereas VE‐cadherin did not differ significantly between groups (time 0 in Figure 1). In adjusted bootstrap quantile regression analyses including BMI and smoking status as covariates, the baseline differences remained significant for both ZO‐1 (adjusted median difference = 25.6, 95% CI 20.1–31.1; *p* < 0.001) and Claudin‐5 (adjusted median difference = 38.6, 95% CI 34.5–42.7; *p* < 0.001), while no significant difference was observed for VE‐cadherin (adjusted median difference = 1.7, 95% CI −0.7–4.1; *p* = 0.165). Notably, in AP patients, the serum levels of both ZO‐1 and Claudin‐5 were significantly decreased after six months of treatment and even more at 12 months post‐treatment compared with baseline, reaching the levels of the control group for ZO‐1 and Claudin‐5 (time 6 and 12 in Figure 1). Changes differed significantly between groups. At 12 months, for ZO‐1, the median change was +0.9 in controls and −17.0 in the AP group (Mann–Whitney *U* test: *z* = 5.643, *p* < 0.001; effect size *r* = 0.84), and this difference remained significant after adjustment for BMI and smoking status (adjusted median difference in change = −16.2, 95% CI −24.0 to −8.4; *p* < 0.001). Similarly, for Claudin‐5, the median change was −1.0 in controls and −34.8 in the AP group (*z* = 5.734, *p* < 0.001; *r* = 0.85), and the between‐group difference remained significant after adjustment for BMI and smoking status (adjusted median difference in change = −33.6, 95% CI −38.6 to −28.6; *p* < 0.001).

**FIGURE 1:**
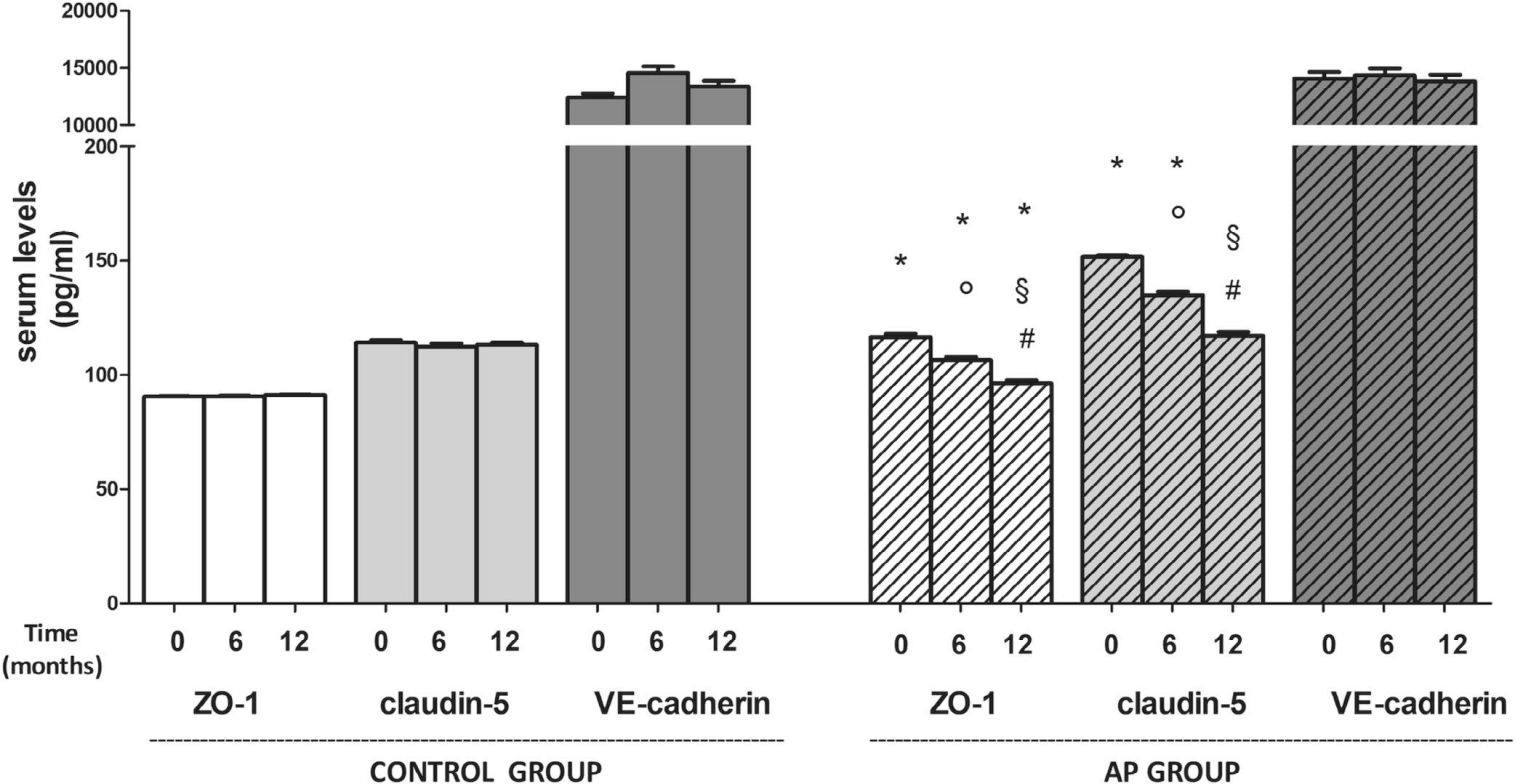
Serum ZO‐1, Claudin‐5, and VE‐cadherin levels were measured in healthy subjects (control group) and in patients with apical periodontitis (AP group) at baseline (0 months), 6 months, and 12 months post‐treatment. *p < 0.0001 AP group vs. control group at the same time, °*p* < 0.0001 AP group at time 6 vs. AP group at time 0, ^#^ *p* < 0.0001 AP group at time 12 vs. AP group at time 0, ^§^ *p* < 0.00001 AP group at time 12 vs. AP group at time 6.

Next, we explored the correlation between serum levels of pro‐inflammatory cytokines (IL‐1β, IL‐6, IL‐8, TNF‐α) and levels of ZO‐1, Claudin‐5 and VE‐cadherin in the AP group. In the unadjusted analysis, baseline ZO‐1 showed moderate positive correlations with IL‐1β (*ρ* = 0.47) and IL‐6 (*ρ* = 0.35) in sera of the AP group. However, these associations did not remain statistically significant after Bonferroni correction for multiple testing and were therefore interpreted as exploratory. No other significant correlations were observed.

Since we found increased levels of Claudin‐5 and ZO‐1 in patients’ serum, which are proteins associated with barrier damage, we decided to test whether systemic inflammation could contribute to in vitro endothelial damage and consequent alterations in vascular permeability. Therefore, we used an in vitro model of microvascular endothelial blood–brain barrier to verify the biological activity of sera on barrier integrity and cellular permeability. The study evaluated whether serum from patients with AP, containing higher levels of cytokines compared with control sera (Multari et al. 2024), would impair the barrier function and whether successful RCT, which was associated with decreased serum levels of pro‐inflammatory cytokines, as demonstrated, could promote the restoration of membrane integrity and permeability in a human cellular model.

For this purpose, hCMEC/D3 cells were pre‐incubated with serum from control subjects and of AP patients, both before and after treatment. Following 24 or 48 h, BBB integrity was evaluated by assessing the permeability to DEX and by measuring TEER.

To validate the experimental system, hCMEC/D3 untreated cells (ctrl) and hCMEC/D3 incubated with IL‐6, a well‐known permeability inducer, were tested. IL‐6 significantly increased permeability values (^§^ *p* < 0.001), indicating a clear effect on cellular function (Figure 2). Then, hCMEC/D3 cells were co‐cultured in the absence or presence of sera from AP and control groups. Dextran passage was significantly increased (*p < 0.0001) in cells pretreated with sera from patients with AP compared with those incubated with sera from the control group (t0 of the AP group compared with t0 of the control group in Figure 2). Notably, permeability decreased in cells cultured with sera from the AP group at 6 and 12 months post‐treatment (°*p* < 0.001 and ^#^ *p* < 0.001, respectively) (Figure 2).

**FIGURE 2:**
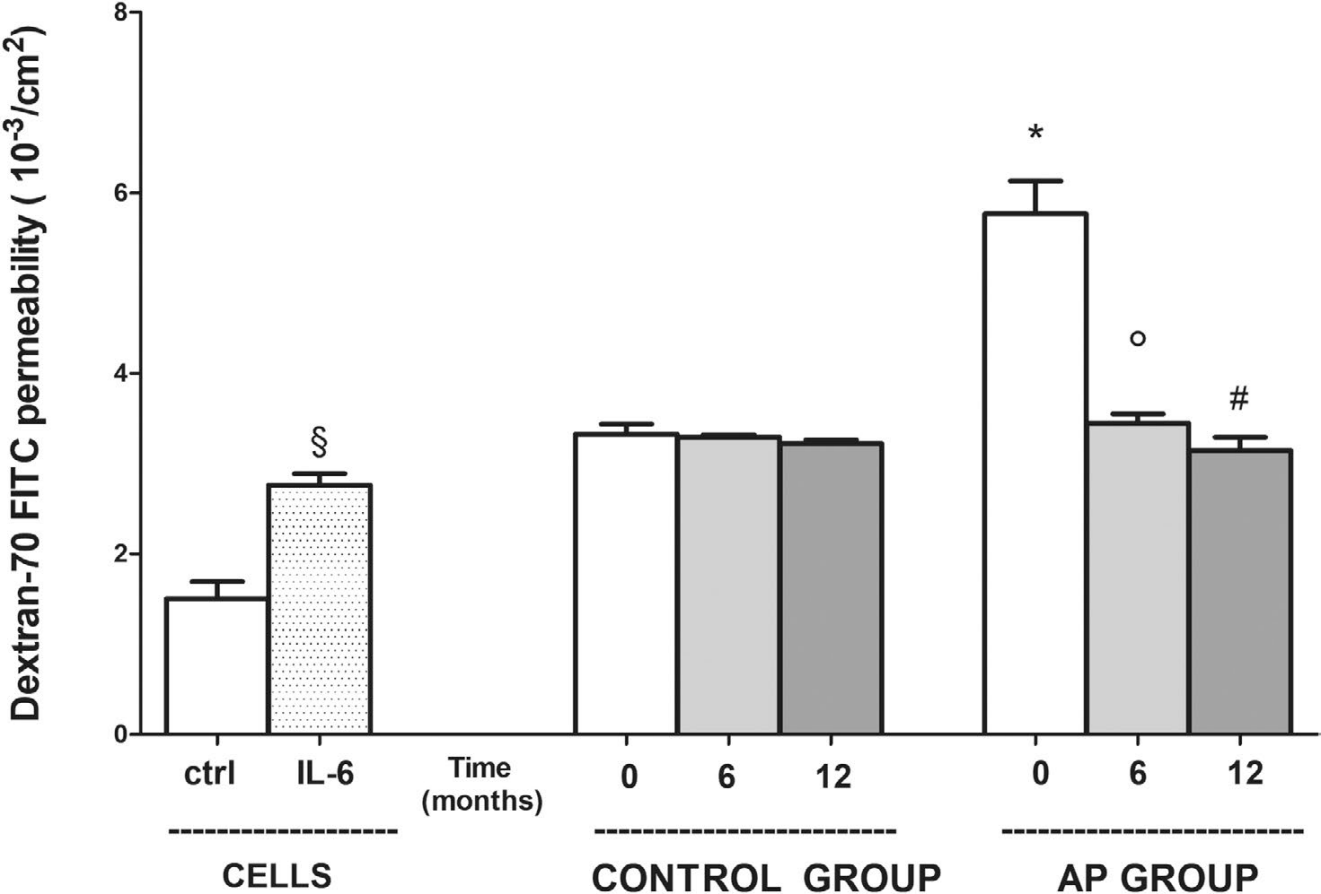
Dextran 70‐fluorescein isothiocyanate permeability assay was used to analyze the permeability of the in vitro BBB model. hCMEC/D3 cells were stimulated for 48 h with sera of healthy subjects (control group) or with sera collected from patients with apical periodontitis (AP group) at baseline (time 0), and at 6 and 12 months post‐treatment. hCMEC/D3 untreated cells (ctrl) and hCMEC/D3 incubated with IL‐6, used as a well‐known permeability inducer, were tested (^§^ *p* < 0.001). Data are presented as mean ± SEM (*n* = 4). *p < 0.0001 AP vs. control group at time 0, °*p* < 0.001 AP group at time 6 vs. AP group at time 0, ^#^ *p* < 0.001 AP group at time 12 vs. AP group at time 0.

The TEER value of the BBB monolayer, under basal condition before serum exposure, ranged from 28 to 38 Ω cm^2^, confirming both the formation of a reliable and fully developed barrier and the functional integrity of the BBB monolayer. To ensure the validity of the experimental model in terms of TEER measurements, hCMEC/D3 untreated cells (ctrl) and hCMEC/D3 stimulated with IL‐6 were analysed. IL‐6 significantly decreased TEER values (*p* < 0.001) (Figure 3), indicating a detectable effect on BBB integrity. After incubation with sera from the AP group at time 0, the TEER values of BBB monolayer decreased compared with the measure of control group (*p < 0.001), whereas they increased and returned to values similar to control group when the incubation was carried out with sera from cured patients (AP group at 6 and 12 months post‐treatment) (°*p* < 0.01 and ^#^ *p* < 0.001) (Figure 3).

**FIGURE 3:**
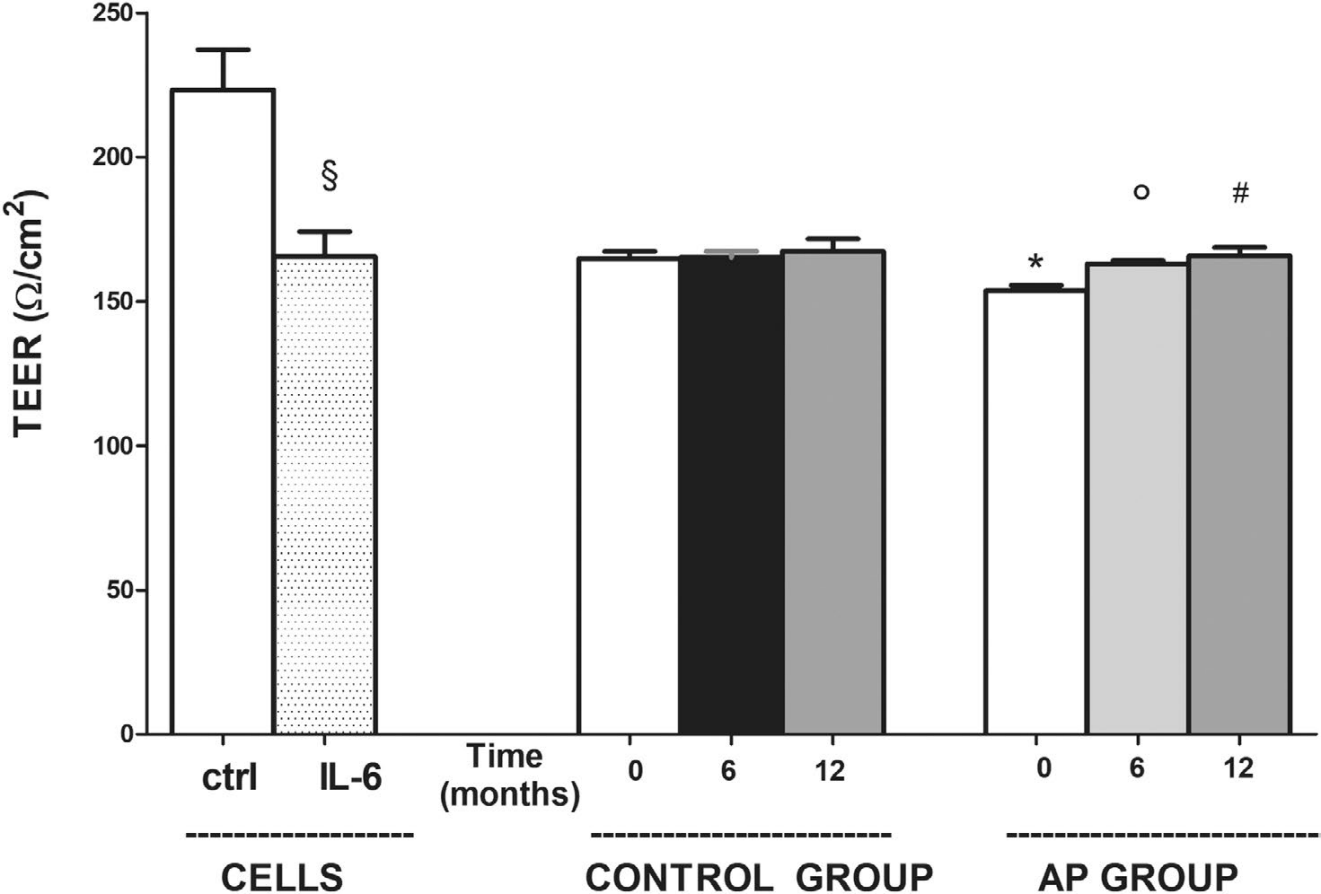
TEER values were measured in hCMEC/D3 cells, treated for 24 h with sera obtained from healthy subjects (control group) or from patients with apical periodontitis (AP group) at baseline (t0), 6 months and 12 months post‐treatment. hCMEC/D3 untreated cells (ctrl) and hCMEC/D3 incubated with IL‐6, used as a well‐known permeability inducer, were tested (^§^ *p* < 0.001). Data are presented as means + SEM (*n* = 4). *p < 0.001 AP vs. control group at time 0, °*p* < 0.01 AP group at time 6 vs. AP group at time 0, ^#^ *p* < 0.001 AP group at time 12 vs. AP group at time 0.

Since we demonstrated that ZO‐1 and Claudin‐5 were released into serum during inflammation, and that their level decreased after endodontic treatment, we sought to verify whether the increase in serum levels is accompanied by decreased expression in barrier tissues. For this reason, we investigated the presence of the two proteins in our in vitro BBB model stimulated with control and pathological sera. The expression and localization of the two TJ proteins were analysed by immunofluorescence, and images were acquired on a confocal microscope, as shown in Figure 4. While we could not detect changes in Claudin‐5 levels due to its weak expression, the fluorescent signal specific for ZO‐1 remained stable when cells were treated with control sera, but decreased markedly after incubation with sera from untreated patients (t0). Interestingly, the endodontic treatment restored expression levels back to a fluorescence level similar to the control (t6 and t12).

**FIGURE 4:**
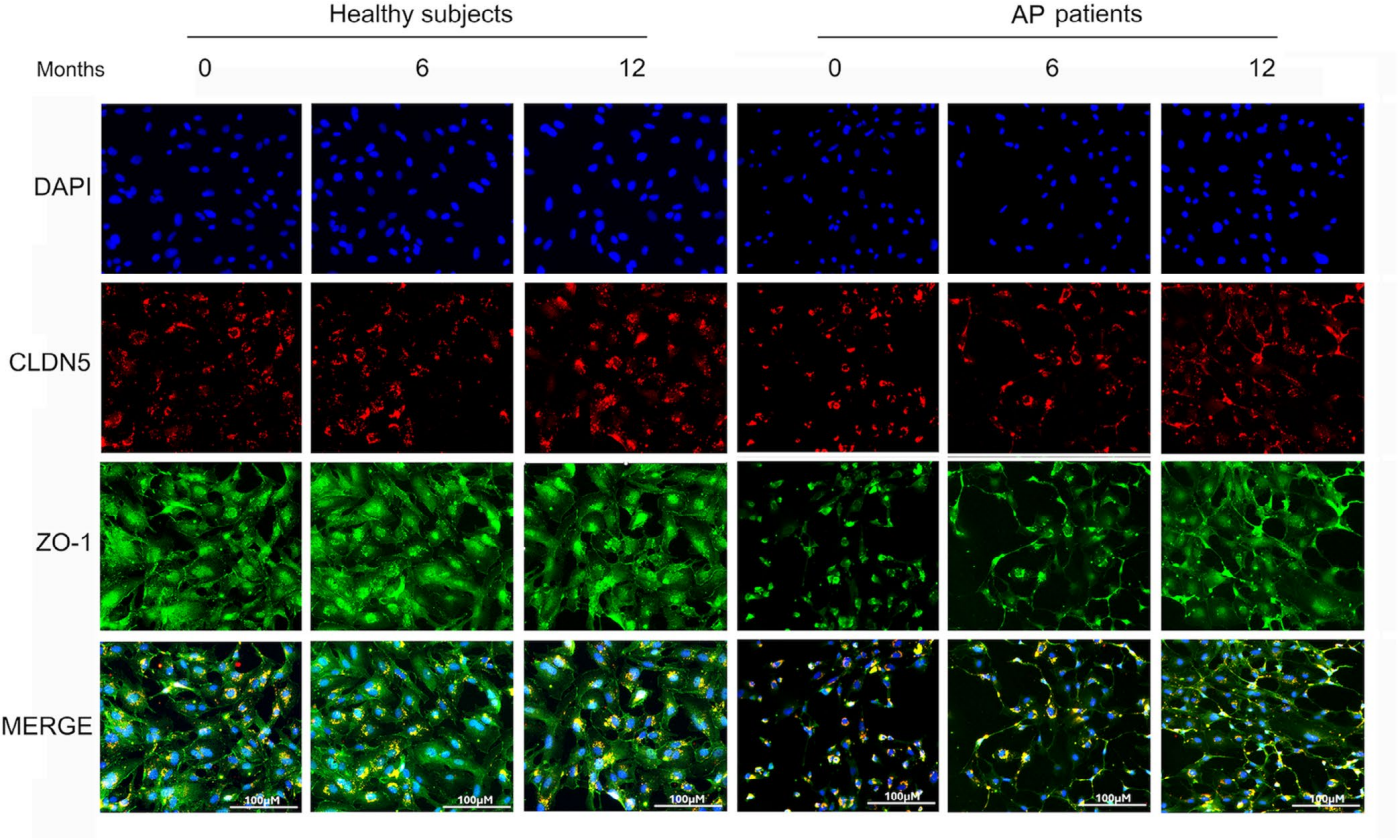
In vitro expression of TJ proteins by immunofluorescence. Cells were treated for 24 h with sera obtained from healthy subjects (control group) or from patients with apical periodontitis (AP patients) at baseline (t0), 6 months and 12 months post‐treatment. Cells were fixed with paraformaldehyde, stained for Claudin‐5 (CLDN5, red), Zonula Occludens‐1 (ZO‐1, green) expression, and nuclei (DAPI staining, blue), and analysed by confocal microscopy. Their overlapping (MERGE) was used to show colocalization of proteins at TJ. Scale bar: 100 μM.

## 4 Discussion

Our previous work (Multari et al. 2024) has shown that AP may contribute to systemic inflammation, evidenced by elevated baseline serum levels of IL‐1β, IL‐6, IL‐8, and TNF‐α in patients with AP compared with healthy controls. Importantly, these cytokine levels can be reduced by RCT (Multari et al. 2024), as also reported in a systematic review with meta‐analysis (Jakovljevic et al. 2025) where the presented findings imply that patients who undergo effective endodontic treatment may benefit in preventing a low‐grade inflammatory burden and the development of further complications related to a primary systemic disease.

Systemic inflammation can alter barrier permeability promoting the passage of molecules and causing tissue damage. Under inflammatory conditions, both epithelial and endothelial target cells are exposed to a variety of cytokines, leading to increased paracellular permeability (Capaldo and Nusrat 2009); (Capaldo et al. 2014) the consequent leakage of the inflammatory mediators and other molecules into the surrounding tissues triggers events that precede the disease and lead to the enhanced intestinal permeability both in IBD (Caviglia et al. 2019); (Hellenthal et al. 2022) and in insulin resistance (Vermette et al. 2018) and obesity (Wood Heickman et al. 2020); (Katz and Rotstein 2021).

ZO‐1 and Claudin‐5 proteins (Fan et al. 2023) present in the tight junction are an index of destabilization of the epithelial/endothelial barriers. In liver diseases, serum zonulin levels correlate with increased hepatic permeability and can predict the severity of cirrhosis and liver failure (Voulgaris et al. 2021). Serum zonulin levels are also associated with excessive permeability of other organs, such as adipose tissue, lung, and skin, although the initial factor that activates increased zonulin secretion in these tissues has not yet been identified (Humphreys 2020).

With regard to endothelial barrier function, numerous studies have demonstrated that BBB permeability increases in response to many pro‐inflammatory stimuli, and that Claudin‐5 downregulation plays a role in this process (Greene et al. 2019). Indeed, among TJ proteins, Claudin‐5 and occludin, which normally restrict paracellular movement of molecules across the BBB, are disrupted in neuro‐inflammatory diseases (Liebner et al. 2018). (Versele et al. 2022) reported that in a human brain‐like endothelial cell monolayer cytokines like IL‐1β and TNF‐α can modulate blood–brain barrier permeability by respectively decreasing ZO‐1 protein level and VE‐cadherin mRNA level, and increasing Claudin‐5 protein level. Indeed, in response to pro‐inflammatory cytokines such as IL‐1β or TNF‐α, cells may attempt to compensate for junctional disruption by upregulating Claudin‐5 expression. However, this compensation may be insufficient or dysfunctional, so barrier permeability still continues to increase (Versele et al. 2022).

To date, there is no scientific evidence to support a causal effect of AP on the onset of altered endothelial permeability. Our previous results showed an association between the systemic inflammatory component of AP and the beneficial impact of endodontic treatment on inflammatory factors. Therefore, in the present study, we investigated whether periapical inflammation can modulate endothelial permeability, and tested the effects of endodontic treatment on barrier function, using an in vitro model of human brain microvascular endothelial cells. For the first time, we observed that patients with AP have higher baseline serum levels of ZO‐1 and Claudin‐5 compared with control subjects. Notably, successful treatment of apical lesions was associated with reduced serum levels of ZO‐1 and Claudin‐5 levels. Conversely, VE‐cadherin did not show significant differences between the AP group and the control group. These results suggest that the BBB lost barrier integrity in the presence of inflammatory cytokines and that endodontic treatment restored barrier integrity, although other mediators in addition to cytokines may also be involved.

In our study, unadjusted correlation analysis showed moderate positive associations between ZO‐1 and IL‐1β and between ZO‐1 and IL‐6 at baseline. However, these associations did not remain statistically significant after correction for multiple testing and should therefore be interpreted with caution as exploratory findings. Overall, the data are consistent with a plausible link between localized oral inflammation and systemic endothelial dysfunction, but they do not allow define conclusions regarding a single cytokine‐mediated mechanism. ZO‐1 is a critical scaffold protein maintaining tight junction structure and function; thus, its modulation by cytokines may contribute to increased permeability and barrier dysfunction (Matter and Balda 2003). The lack of correlation with Claudin‐5 and VE‐cadherin suggests that other regulatory mechanisms or factors may control these junctional proteins in AP or that their expression in serum may not directly reflect endothelial changes. Importantly, the main between‐group differences for ZO‐1 and Claudin‐5 at baseline, as well as the between‐group differences in 12‐month change, remained significant after adjustment for BMI and smoking status, supporting the robustness of these findings. We cannot definitively determine the origin of the permeability proteins in AP sera.

Few previous studies have investigated the influence of inflammatory cytokines on barrier disruption. For example, (Yang et al. 2016) showed that in an in vitro model of the human BBB, exposure to IL‐1β affects TJ proteins, including ZO‐1, resulting in BBB disruption and increased permeability to FITC‐dextran. For the first time, our study provides evidence that sera from AP patients can modulate barrier‐related properties in a human BBB‐like endothelial model.

In fact, we set up a competent human BBB model incubated for 48 h with each serum from the control group or the AP group before endodontic treatment (time 0), and at 6 and 12 months after RCT. In this in vitro BBB model, we carried out functional and protein expression assays. To test barrier function, the permeability to DEXT was used as a parameter of TJ permeability since it cannot cross the BBB if TJ complexes are in a competent state (Digiovanni et al. 2024). BBB cells incubated with serum from AP patients at 0 months showed a greater apical‐to‐basolateral permeability to DEXT compared with the BBB monolayer incubated with serum from control subjects at 0 months. As dextran passage was significantly increased in cells pretreated with sera from patients with AP compared with those incubated with sera from the control group (t0 of AP group compared with t0 of the control group), the observation suggests that sera from the AP group induced a significant modification of cellular or paracellular flux across the cell monolayer. Endodontic cure led to the restoration of impermeability to dextran, as demonstrated by the decreased transport across cells cultured in sera from the AP group at 6 and 12 months post‐treatment.

In addition, TEER values of BBB monolayer were lower in patients with AP than in control patients indicating the loss of BBB integrity, suggesting the impairment of BBB barrier integrity in the presence of pathological serum and that the endodontic treatment mitigated the damage. Moreover, the expression of the main barrier protein ZO‐1 was reduced in barrier cells treated with sera from AP patients, whereas the recovery of ZO‐1 protein after endodontic treatment was demonstrated by immunofluorescence. This is really novel evidence that sera from AP patients can disrupt endothelial barrier function, and this study opens new interesting avenues for research on AP.

In vitro, we investigated the effect of sera on a brain barrier model. The most clinically relevant discovery of this study is that, in vitro, the barrier dysfunction induced by pathological sera can be reverted by endodontic cure, both at 6 and 12 months after therapy. In fact, both functional experiments and protein expression analysis revealed that permeability, integrity, and expression of barrier proteins were restored when BBB cells were incubated with sera from treated patients. This novel finding aligns well with the observed decrease in serum levels of inflammatory cytokines and the reduced release of TJ proteins in sera from healed patients. Further studies should explore other models, such as the intestinal and liver barriers, which may show a similar effect and better highlight the systemic impact of AP as a cause of multi‐organ diseases with an inflammatory basis.

This study has several strengths, including its prospective longitudinal design, the use of a well‐characterized control group, repeated follow‐up after endodontic treatment, and the combined assessment of clinical data with in vivo and in vitro analyses, providing exploratory insights into the systemic effects associated with apical periodontitis.

Some limitations should, however, be acknowledged. First, the relatively limited sample size did not allow stratified analyses for potentially relevant variables, and although the cohort had originally been established on the basis of a previous power calculation for cytokine‐related analyses, no dedicated a priori sample size calculation was performed for the permeability‐related proteins evaluated here. Second, although we adjusted the main analyses for BMI and smoking status and applied strict exclusion criteria to reduce major sources of systemic inflammation, residual confounding from unmeasured variables and intercurrent health conditions cannot be ruled out. Third, despite correction for multiple testing in repeated pairwise comparisons and correlation analyses, the possibility of type I error cannot be completely excluded. Moreover, because different endodontic sealers were used during treatment protocol as part of routine clinical practice performed by a single experienced operator following a standardised treatment protocol, the potential influence of sealer‐related biological variability on systemic outcomes was not specifically assessed and cannot be completely excluded. In addition, the radiographic lesion classification, which was based on combined clinical and radiographic criteria rather than a standardised scoring index, should be considered when interpreting the findings. Finally, the hCMEC/D3 cell line is a useful tool for studying endothelial mechanisms but represents a simplified in vitro model that does not fully reproduce the complexity of the blood–brain barrier in vivo. Thus, the findings should be considered preliminary and not potentially translatable to clinical endothelial dysfunction. Further validation in more physiologically relevant models, such as advanced co‐culture systems, organ‐on‐chip platforms, or in vivo observation studies, is required. A more advanced model has recently been developed by the authors to support future investigations (Digiovanni et al. 2024).

Taken together, the results of this work and the previous evidence published by our research group, underline how the treatment of AP is of fundamental importance not only for the single tissue but for the general health of the patient and may reduce a chronic inflammatory state which could otherwise be detrimental.

## Author Contributions

Loredana Bergandi: conceptualization, methodology, investigation, formal analysis, writing – original draft; Mario Alovisi: conceptualization, methodology, investigation, formal analysis, writing – original draft; Elena Tachis: methodology, investigation, writing – original draft; Nicola Scotti: review and editing; Andrea Baldi: review and editing; Iris Chiara Salaroglio: investigation, writing – original draft; Sabrina Digiovanni: investigation; Lorena Charrier: formal analysis, validation, review and editing; Francesca Silvagno: review and editing; Damiano Pasqualini: project administration, conceptualization, supervision, writing – original draft, review and editing.

## Funding

This work was supported by Ministero dell’Istruzione, dell’Università e della Ricerca, PNC 0000001 D3 4 Health project.

## Ethics Statement

The observational study was designed and carried out with the approval of the *Città della Salute e della Scienza Ethical Committee* (prot. nr. 0094485; CS2/510 and Clinical Trial n. NCT07292363) and conducted in accordance with the latest update of the Helsinki Declaration.

## Conflicts of Interest

The authors declare no conflicts of interest.

## Data Availability

The data that support the findings of this study are available from the corresponding author upon reasonable request.
